# Synthesis, crystal structure and Hirshfeld surface analysis of 2-(perfluoro­phen­yl)acetamide in comparison with some related compounds

**DOI:** 10.1107/S2056989021013359

**Published:** 2022-01-01

**Authors:** Anton P. Novikov, Alexey A. Bezdomnikov, Mikhail S. Grigoriev, Konstantin E. German

**Affiliations:** a Peoples’ Friendship University of Russia (RUDN University), 6 Miklukho-Maklaya, St, 117198, Moscow, Russian Federation; b Frumkin Institute of Physical Chemistry and Electrochemistry Russian, Academy of Sciences, 31 Leninsky Prospekt bldg 4, 119071 Moscow, Russian Federation

**Keywords:** crystal structure, hydrogen bonds, Hirshfeld surface analysis, acetamide, perfluoro­phen­yl

## Abstract

The mol­ecular and crystal structures of the title compound, C_8_H_4_F_5_NO, were examined by single-crystal X-ray diffraction and Hirshfeld surface analysis. The title compound was synthesized by a new method at the interface of aqueous solutions of LiOH and penta­fluoro­phenyl­aceto­nitrile. In the crystal, hydrogen bonds and π–halogen inter­actions connect the mol­ecules into double layers. Analysis of the Hirshfeld surface showed that the most important contributions to the crystal packing are made by F⋯F (30.4%), C⋯F/F⋯C (22.9%), O⋯H/H⋯O (14.9%), H⋯F/F⋯H (14.0%) and H⋯H (10.2%) contacts. The Hirshfeld surfaces of analogues of the title compound were compared and the effect of perfluorination on the crystal packing was shown.

## Abstract 2

The title compound was synthesized by a new method at the inter­phase of aqueous solutions of LiOH and penta­fluoro­phenyl­aceto­nitrile. In the crystal, hydrogen bonds and π–halogen inter­actions connect the mol­ecules into double layers. The Hirshfeld surfaces of analogues of the title compound were compared and the effect of perfluorination on the crystal packing was shown.

## Chemical context

The development of effective methods for the formation of an amide bond CON*R*_2_ is of great importance because of the high synthetic value of amides, their industrial applications and pharmacological inter­est (Massolo *et al.*, 2020 ▸). The addition of functional groups such as –F, –Cl *etc*. can improve the catalytic or biological activity of the corresponding coordin­ation compounds (Naumann, 2003 ▸). 
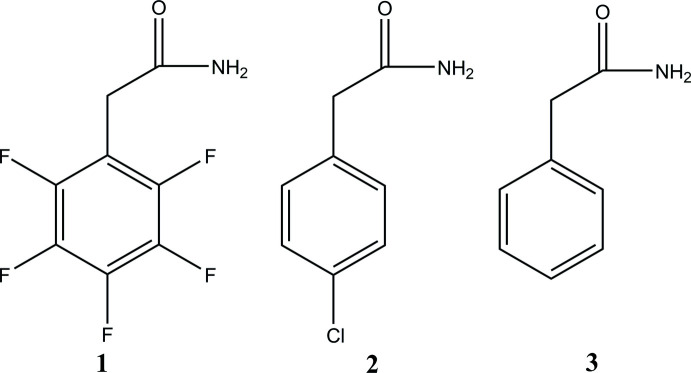


The title compound was previously obtained (Barbour *et al.*, 1961 ▸) using 2,3,4,5,6-penta­fluoro­benzoic acid as the starting compound, but its crystal structure was not studied. In this work, we have synthesized 2–(perfluoro­phen­yl) acetamide, **1**, from penta­fluoro­phenyl­aceto­nitrile and determined its crystal structure. We have analysed the Hirshfeld surface of this compound and compared it with those of 2-(4-chloro­phen­yl) acetamide, **2** (OCETAT; Ma *et al.*, 2011 ▸) and 2-phenyl­acetamide, **3** (SAWHAC; Skelton *et al.*, 2017 ▸).

## Structural commentary

**Figure 1:**
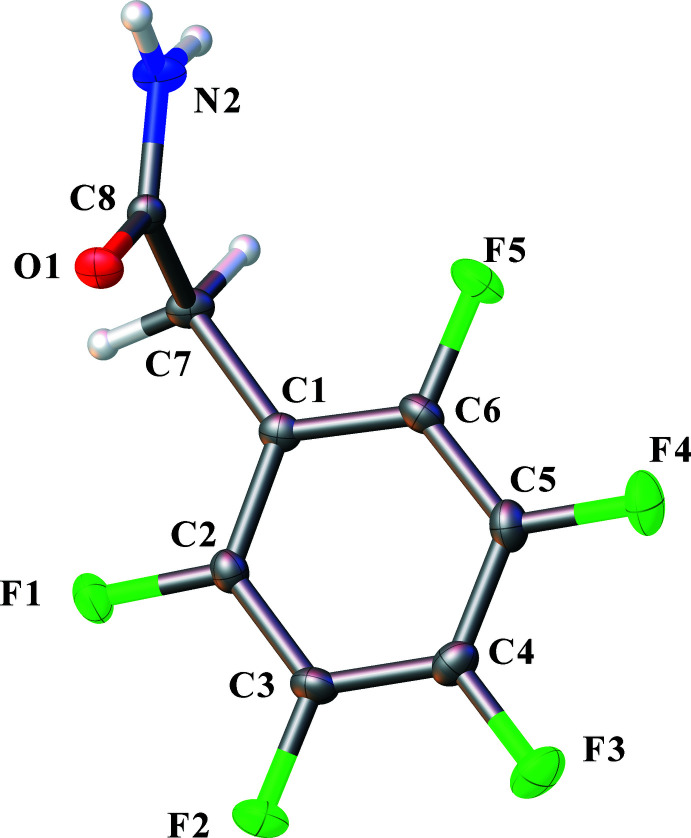
Mol­ecular structure of the title compound, including atom labelling. Displacement ellipsoids are drawn at the 50% probability level.

The title compound crystallizes in the space group *P*2_1_/*c* with four mol­ecules in the unit cell. All H atoms in the phenyl ring are replaced by fluorine atoms. The asymmetric unit is illustrated in Fig. 1 ▸. Carbon atom C7 is in the plane of the imidazole ring. The acetamide group is twisted relative to the Ph-ring with a C2—C1—C7—C8 torsion angle of 107.61 (14)°. This angle is larger than in the 3-chloro-4-hy­droxy­phenyl acetamide (Davis *et al.*, 2005 ▸). Torsion angles C8—C7—C1—C6 and C1—C7—C8—N2 are −74.35 (15) and 134.77 (12)°, respectively. This conformation is probably a consequence of inter­molecular hydrogen bonds and steric factors.

## Supra­molecular features

**Figure 2:**
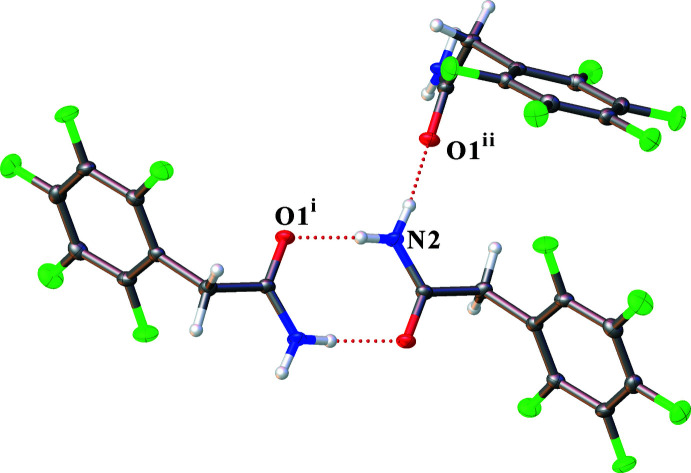
View showing hydrogen bonds in **1**. [Symmetry codes: (i) −*x*, −*y* + 2, −*z* + 1; (ii) *x*, −*y* + 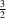 , *z* − 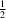 .]

The hydrogen-bond system is shown in Fig. 2 ▸. In the structure, there are three hydrogen bonds. Two relatively strong hydrogen bonds are formed between the amino group and the oxygen atom of the carbonyl group. The shortest hydrogen bond N2—H2*B*⋯O1^ii^ [symmetry code: (ii) *x*, −*y* + 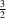 , *z* − 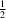 ] is 2.8795 (14) Å (Table 1 ▸). The structure also contains one short contact of the type C—H⋯F with a C7—H7*B*⋯F1^iii^ [symmetry code: (iii) *x*, −*y* + 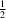 , *z* − 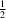 ] distance of 3.3764 (15) Å, but this cannot be called a hydrogen bond (Howard *et al.*, 1996 ▸). The structure contains a short contact between the fluorine atoms F4 and F4^iv^ [symmetry code: (iv) −*x* + 1, −*y* + 2, −*z* + 1], whose length of 2.6649 (18) Å is shorter then the sum of the van der Waals radii (Mantina *et al.*, 2009 ▸). However, according to the recommendations of Cavallo *et al.* (2016 ▸), it cannot be considered to be a halogen bond.

**Figure 3:**
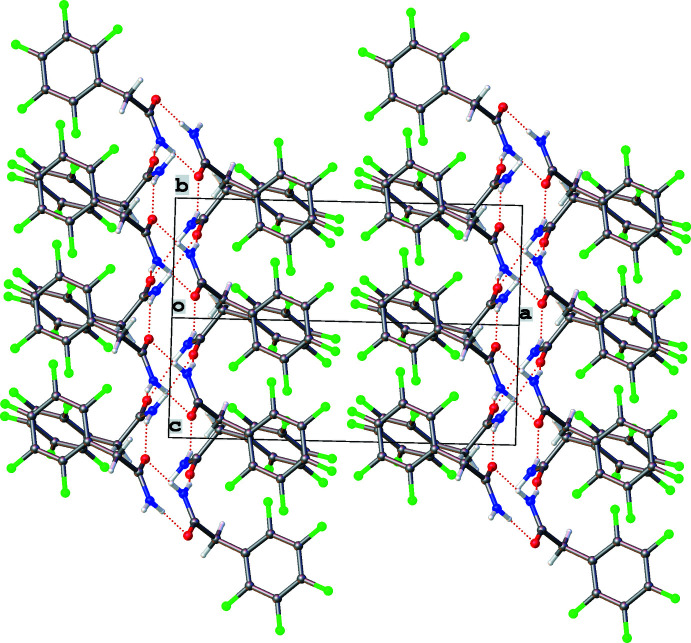
Crystal packing of **1** showing the double layers.

The crystal packing can be represented as layered, as shown in Fig. 3 ▸. The hydrogen bonds bind mol­ecules inside double layers parallel to (100). This type of packing can be found in the structure of 5,5-di­chloro-6-hy­droxy­dihydro­pyrimidine-2,4(1*H*,3*H*)-dione (Novikov *et al.*, 2020 ▸).

## Hirshfeld surface analysis

**Figure 4:**
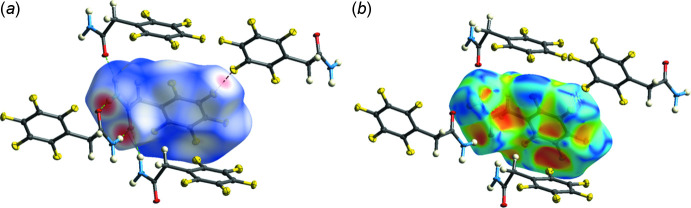
Hirshfeld surface mapped over (*a*) *d*_norm_ and (*b*) shape-index to visualize the inter­actions in the title compound.

**Figure 5:**
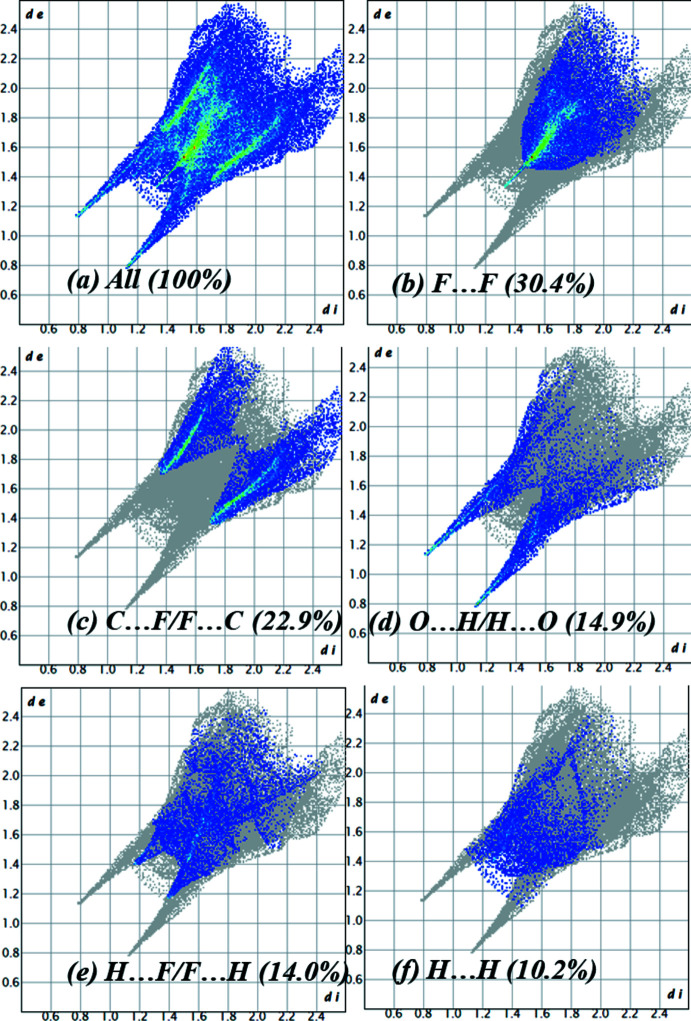
(*a*) The full two-dimensional fingerprint plot for the title compound, together with those delineated into (*b*) F⋯F, (*c*) C⋯F/F⋯C, (*d*) O⋯H/H⋯O, (*e*) H⋯F/F⋯H and (*f*) H⋯H contacts.

*Crystal Explorer 21* was used to calculate the Hirshfeld surfaces and two-dimensional fingerprint plots (Spackman *et al.*, 2021 ▸). The donor–acceptor groups are visualized using a standard (high) surface resolution and the *d*_norm_ surfaces are mapped over a fixed colour scale of −0.542 (red) to 1.121 (blue) a.u., as illustrated in Fig. 4 ▸ *a*. The most important hydrogen bonds, N2—H2*A*⋯O1^i^ are N2—H2*B*⋯O1^ii^, are shown by red spots on the surface. A weak red spot may indicate the presence of a π-inter­action between the C atom of the ring and the F atom of another ring. There are no π-stacking inter­actions of the mol­ecules, which can be seen from the absence of characteristic triangles in Fig. 4 ▸ *b*. However, a bright spot on the shape-index surface may indicate the presence of a π-halogen inter­action. The overall two-dimensional fingerprint map for the title compound is shown in Fig. 5 ▸ *a*. The fingerprint plots show that the F⋯F contacts (30.4%) make the largest contribution to the overall packing of the crystal. Contacts of the C⋯F/F⋯C type also make a significant contribution (22.9%). This can also be related to the presence of a π—F inter­action in the structure. The short distances C1⋯F2^iii^ [symmetry code: (iii) *x*, −*y* + 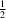 , *z* − 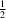 ] and C4⋯F5^v^ [symmetry code: (v) *x*, −*y* + 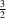 , *z* + 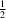 ], which are 3.079 (2) and 3.110 (2) Å, respectively, confirm this fact (Novikov *et al.*, 2021 ▸; Zhuo *et al.*, 2014 ▸). The O⋯H/H⋯O and H⋯F/F⋯H hydrogen bonds also make a significant contribution to the Hirshfeld surface area (28.9% in total). In addition, van der Waals inter­actions (H⋯H) contribute 10.2%. The contribution of other contacts is less than 8% in total and is not discussed in this work.

**Figure 6:**
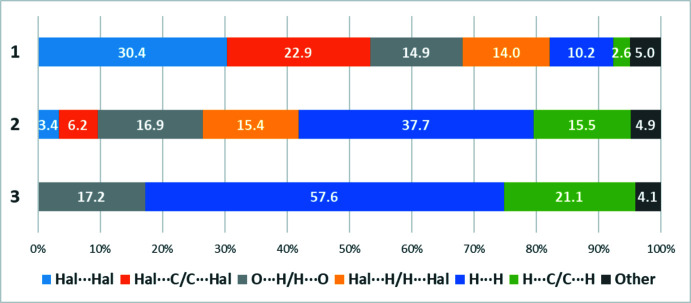
Percentage contributions of contacts to the Hirshfeld surface in the title compound and for related compounds.

An analogous methodology was applied to construction of Hirshfeld surfaces for similar benzenamide derivatives with one Cl substituent and an unsubstituted six-membered ring. The effect of perfluorination is evident from the comparison made in Fig. 6 ▸. On going from the title compound **1** to compound **3**, the halogen bonds disappear. Moreover, if only one hydrogen atom in the ring is replaced by chlorine, the proportion of halogen and π-halogen bonds is significantly reduced. However, in the transition from compound **1** to **3**, the contribution of van der Waals inter­actions increases.

## Database survey

A search in the Cambridge Structural Database (CSD, Version 5.42, update of September 2021; Groom *et al.*, 2016 ▸) gave only a few results for phenyl acetamide. We have found no compound with a fluorine-substituted phenyl ring similar to the title compound. In **3** (SAWHAC; Skelton *et al.*, 2017 ▸), the H atoms in the phenyl ring are not substituted, and this compound crystallizes in space group *C*2/*c* different from **1**. In **2** (OCETAT; Ma *et al.*, 2011 ▸) the hydrogen atom in the *para*-position to the acetamide group is substituted with chlorine. FIXCEV (Davis *et al.*, 2005 ▸) contains a chlorine atom at the *meta-*position and a hydroxo-group at the *para*-position to the amido group. However, as a result of the presence of a hydroxo group, a different system of hydrogen bonds and packing is present in the structure, as is the case in the structure of BHXPAM10 (McMillan *et al.*, 1975 ▸), where there are two bromine atoms and two hydroxo groups.

## Synthesis and crystallization

A saturated aqueous solution of LiOH (0.5 mL, at 298 K) and 2,3,4,5,6-penta­fluoro­phenyl­aceto­nitrile (0.5 mL) were placed in a 1.5 mL screw-neck vial. The closed vial was shaken in a water bath at 383 K until the organic phase turned dark red (30 min). The closed vial with the resulting two-phase system was left for three days at 298 K, and slow growth of the crystal phase at the inter­face of water-2,3,4,5,6-penta­fluoro­phenyl­aceto­nitrile was observed. The obtained crystals were identified as 2-(penta­fluoro­phen­yl)acetamide by X-ray structural analysis.

## Refinement

Crystal data, data collection and structure refinement details are summarized in Table 2 ▸. N- and C-bound H atoms were refined isotropically [*U*_iso_(H) = 1.2*U*_eq_(N,C)].

## Supplementary Material

Crystal structure: contains datablock(s) I. DOI: 10.1107/S2056989021013359/zv2012sup1.cif

Structure factors: contains datablock(s) I. DOI: 10.1107/S2056989021013359/zv2012Isup2.hkl

Supporting information file. DOI: 10.1107/S2056989021013359/zv2012Isup3.cml

CCDC reference: 2128969

Additional supporting information:  crystallographic information; 3D view; checkCIF report

## Figures and Tables

**Table 1:** Hydrogen-bond geometry (Å, °)

*D*—H⋯*A*	*D*—H	H⋯*A*	*D*⋯*A*	*D*—H⋯*A*
N2—H2*A*⋯O1^i^	0.853 (19)	2.062 (19)	2.9120 (15)	174.3 (16)
N2—H2*B*⋯O1^ii^	0.869 (18)	2.053 (19)	2.8795 (14)	158.7 (16)

Symmetry codes: (i) -x, -y+2, -z+1; (ii) x, -y+{\script{3\over 2}}, z-{\script{1\over 2}}.

**Table 2:** Experimental details

Crystal data	
Chemical formula	C_8_H_4_F_5_NO
*M*_r_	225.12
Crystal system, space group	Monoclinic, *P*2_1_/*c*
Temperature (K)	100
*a*, *b*, *c* (Å)	14.4934 (5), 5.8247 (2), 9.7836 (3)
β (°)	90.870 (2)
*V* (Å^3^)	825.83 (5)
*Z*	4
Radiation type	Mo *K*α
μ (mm^−1^)	0.20
Crystal size (mm)	0.2 × 0.15 × 0.06

Data collection	
Diffractometer	Bruker *KAPPA* APEXII area-detector diffractometer
Absorption correction	Multi-scan (*SADABS*; Bruker, 2008 ▸)
*T*_min_, *T*_max_	0.888, 1.000
No. of measured, independent and observed [*I* > 2σ(*I*)] reflections	10007, 2394, 1868
*R*_int_	0.032
(sin θ/λ)_max_ (Å^−1^)	0.703

Refinement	
*R*[*F* ^2^ > 2σ(*F* ^2^)], *wR*(*F* ^2^), *S*	0.036, 0.101, 1.02
No. of reflections	2394
No. of parameters	148
H-atom treatment	Only H-atom coordinates refined
Δρ_max_, Δρ_min_ (e Å^−3^)	0.41, −0.22

Computer programs: *APEX2* and *SAINT* (Bruker, 2008 ▸), *SHELXS* (Sheldrick, 2008 ▸), *SHELXL2018/3* (Sheldrick, 2015 ▸) and *OLEX2* (Dolomanov *et al.*, 2009 ▸).

## supplementary crystallographic information

### Crystal data

**Table:**

C_8_H_4_F_5_NO	*F*(000) = 448
*M**_r_* = 225.12	*D*_x_ = 1.811 Mg m^−^^3^
Monoclinic, *P*2_1_/*c*	Mo *K*α radiation, λ = 0.71073 Å
*a* = 14.4934 (5) Å	Cell parameters from 2651 reflections
*b* = 5.8247 (2) Å	θ = 3.8–30.0°
*c* = 9.7836 (3) Å	µ = 0.20 mm^−^^1^
β = 90.870 (2)°	*T* = 100 K
*V* = 825.83 (5) Å^3^	Fragment, colourless
*Z* = 4	0.2 × 0.15 × 0.06 mm

### Data collection

**Table:**

Bruker KAPPA APEXII area-detector diffractometer	1868 reflections with *I* > 2σ(*I*)
φ and ω scans	*R*_int_ = 0.032
Absorption correction: multi-scan (SADABS; Bruker, 2008)	θ_max_ = 30.0°, θ_min_ = 4.2°
*T*_min_ = 0.888, *T*_max_ = 1.000	*h* = −20→20
10007 measured reflections	*k* = −7→8
2394 independent reflections	*l* = −13→13

### Refinement

**Table:**

Refinement on *F*^2^	Primary atom site location: structure-invariant direct methods
Least-squares matrix: full	Hydrogen site location: difference Fourier map
*R*[*F*^2^ > 2σ(*F*^2^)] = 0.036	Only H-atom coordinates refined
*wR*(*F*^2^) = 0.101	*w* = 1/[σ^2^(*F*_o_^2^) + (0.0514*P*)^2^ + 0.2972*P*] where *P* = (*F*_o_^2^ + 2*F*_c_^2^)/3
*S* = 1.01	(Δ/σ)_max_ < 0.001
2394 reflections	Δρ_max_ = 0.41 e Å^−^^3^
148 parameters	Δρ_min_ = −0.22 e Å^−^^3^
0 restraints	

### Special details

**Table:**

Geometry. All esds (except the esd in the dihedral angle between two l.s. planes) are estimated using the full covariance matrix. The cell esds are taken into account individually in the estimation of esds in distances, angles and torsion angles; correlations between esds in cell parameters are only used when they are defined by crystal symmetry. An approximate (isotropic) treatment of cell esds is used for estimating esds involving l.s. planes.

### Fractional atomic coordinates and isotropic or equivalent isotropic displacement parameters (Å^2^)

**Table:**

	*x*	*y*	*z*	*U*_iso_*/*U*_eq_	
F1	0.18090 (6)	0.17229 (15)	0.56871 (9)	0.0236 (2)	
F5	0.29215 (6)	0.86391 (15)	0.38309 (9)	0.0256 (2)	
F2	0.33786 (6)	0.12487 (16)	0.71473 (9)	0.0277 (2)	
F3	0.47319 (6)	0.44830 (17)	0.69860 (9)	0.0282 (2)	
F4	0.44951 (6)	0.81691 (16)	0.53158 (10)	0.0291 (2)	
C7	0.14592 (9)	0.5453 (2)	0.38358 (13)	0.0164 (2)	
H7A	0.1111 (12)	0.407 (3)	0.3821 (17)	0.020*	
H7B	0.1623 (11)	0.578 (3)	0.2907 (18)	0.020*	
C2	0.24618 (9)	0.3351 (2)	0.55727 (13)	0.0160 (3)	
C4	0.39563 (9)	0.4708 (3)	0.62489 (13)	0.0193 (3)	
C5	0.38321 (9)	0.6590 (2)	0.54069 (14)	0.0190 (3)	
C6	0.30222 (9)	0.6800 (2)	0.46574 (13)	0.0168 (3)	
C1	0.23166 (8)	0.5202 (2)	0.47089 (12)	0.0142 (2)	
C3	0.32681 (9)	0.3079 (2)	0.63280 (13)	0.0181 (3)	
C8	0.08285 (8)	0.7324 (2)	0.43665 (12)	0.0138 (2)	
O1	0.06484 (6)	0.74411 (16)	0.56027 (9)	0.0168 (2)	
N2	0.04797 (8)	0.8763 (2)	0.34425 (11)	0.0178 (2)	
H2A	0.0121 (12)	0.985 (3)	0.3679 (18)	0.021*	
H2B	0.0642 (12)	0.868 (3)	0.2593 (19)	0.021*	

### Atomic displacement parameters (Å^2^)

**Table:**

	*U*^11^	*U*^22^	*U*^33^	*U*^12^	*U*^13^	*U*^23^
F1	0.0232 (4)	0.0218 (4)	0.0259 (4)	−0.0081 (3)	−0.0001 (3)	0.0061 (3)
F5	0.0335 (5)	0.0193 (4)	0.0240 (4)	−0.0024 (3)	0.0013 (3)	0.0094 (3)
F2	0.0301 (5)	0.0281 (5)	0.0247 (4)	0.0023 (4)	−0.0027 (3)	0.0139 (4)
F3	0.0200 (4)	0.0387 (5)	0.0257 (4)	0.0010 (4)	−0.0091 (3)	−0.0006 (4)
F4	0.0252 (4)	0.0281 (5)	0.0340 (5)	−0.0138 (4)	0.0018 (3)	−0.0017 (4)
C7	0.0203 (6)	0.0176 (6)	0.0111 (5)	0.0022 (5)	−0.0017 (4)	−0.0026 (5)
C2	0.0171 (6)	0.0169 (6)	0.0142 (6)	−0.0025 (5)	0.0015 (4)	−0.0001 (5)
C4	0.0171 (6)	0.0254 (7)	0.0153 (6)	0.0018 (5)	−0.0017 (4)	−0.0024 (5)
C5	0.0189 (6)	0.0195 (7)	0.0188 (6)	−0.0055 (5)	0.0026 (5)	−0.0032 (5)
C6	0.0221 (6)	0.0149 (6)	0.0134 (6)	0.0001 (5)	0.0021 (4)	0.0021 (4)
C1	0.0171 (5)	0.0155 (6)	0.0100 (5)	0.0012 (4)	0.0011 (4)	−0.0014 (4)
C3	0.0218 (6)	0.0187 (6)	0.0138 (6)	0.0027 (5)	0.0004 (4)	0.0040 (5)
C8	0.0148 (5)	0.0167 (6)	0.0100 (5)	−0.0013 (4)	−0.0004 (4)	−0.0007 (4)
O1	0.0217 (4)	0.0206 (5)	0.0082 (4)	0.0020 (4)	0.0009 (3)	0.0001 (3)
N2	0.0234 (5)	0.0226 (6)	0.0075 (5)	0.0059 (5)	0.0003 (4)	0.0001 (4)

### Geometric parameters (Å, º)

**Table:**

F1—C2	1.3454 (15)	C2—C3	1.3823 (18)
F5—C6	1.3485 (15)	C4—C5	1.381 (2)
F2—C3	1.3421 (15)	C4—C3	1.3795 (19)
F3—C4	1.3328 (15)	C5—C6	1.3800 (18)
F4—C5	1.3343 (15)	C6—C1	1.3844 (18)
C7—H7A	0.949 (18)	C8—O1	1.2430 (15)
C7—H7B	0.962 (17)	C8—N2	1.3272 (17)
C7—C1	1.5043 (17)	N2—H2A	0.853 (19)
C7—C8	1.5192 (18)	N2—H2B	0.869 (18)
C2—C1	1.3841 (18)		
			
H7A—C7—H7B	107.0 (14)	F5—C6—C5	118.21 (12)
C1—C7—H7A	111.2 (10)	F5—C6—C1	118.86 (12)
C1—C7—H7B	110.0 (10)	C5—C6—C1	122.93 (12)
C1—C7—C8	111.83 (10)	C2—C1—C7	122.62 (12)
C8—C7—H7A	106.9 (10)	C2—C1—C6	116.14 (11)
C8—C7—H7B	109.7 (10)	C6—C1—C7	121.22 (12)
F1—C2—C1	119.95 (11)	F2—C3—C2	120.16 (12)
F1—C2—C3	117.70 (11)	F2—C3—C4	120.01 (12)
C3—C2—C1	122.34 (12)	C4—C3—C2	119.83 (12)
F3—C4—C5	120.14 (12)	O1—C8—C7	120.54 (11)
F3—C4—C3	120.43 (12)	O1—C8—N2	123.01 (12)
C3—C4—C5	119.43 (12)	N2—C8—C7	116.44 (11)
F4—C5—C4	119.96 (12)	C8—N2—H2A	120.7 (12)
F4—C5—C6	120.71 (12)	C8—N2—H2B	120.6 (12)
C6—C5—C4	119.32 (12)	H2A—N2—H2B	118.6 (16)
			
F1—C2—C1—C7	−2.11 (19)	C5—C4—C3—F2	−179.69 (12)
F1—C2—C1—C6	179.76 (11)	C5—C4—C3—C2	−0.4 (2)
F1—C2—C3—F2	−0.42 (19)	C5—C6—C1—C7	−177.73 (12)
F1—C2—C3—C4	−179.75 (12)	C5—C6—C1—C2	0.43 (19)
F5—C6—C1—C7	1.67 (18)	C1—C7—C8—O1	−46.31 (16)
F5—C6—C1—C2	179.83 (11)	C1—C7—C8—N2	134.77 (12)
F3—C4—C5—F4	1.0 (2)	C1—C2—C3—F2	−179.69 (12)
F3—C4—C5—C6	−179.62 (12)	C1—C2—C3—C4	1.0 (2)
F3—C4—C3—F2	−0.2 (2)	C3—C2—C1—C7	177.14 (12)
F3—C4—C3—C2	179.08 (12)	C3—C2—C1—C6	−1.00 (19)
F4—C5—C6—F5	0.16 (19)	C3—C4—C5—F4	−179.60 (12)
F4—C5—C6—C1	179.55 (12)	C3—C4—C5—C6	−0.2 (2)
C4—C5—C6—F5	−179.26 (12)	C8—C7—C1—C2	107.61 (14)
C4—C5—C6—C1	0.1 (2)	C8—C7—C1—C6	−74.35 (15)

### Hydrogen-bond geometry (Å, º)

**Table:**

*D*—H···*A*	*D*—H	H···*A*	*D*···*A*	*D*—H···*A*
N2—H2*A*···O1^i^	0.853 (19)	2.062 (19)	2.9120 (15)	174.3 (16)
N2—H2*B*···O1^ii^	0.869 (18)	2.053 (19)	2.8795 (14)	158.7 (16)

Symmetry codes: (i) −*x*, −*y*+2, −*z*+1; (ii) *x*, −*y*+3/2, *z*−1/2.
